# Wild Bornean orangutans experience muscle catabolism during episodes of fruit scarcity

**DOI:** 10.1038/s41598-021-89186-4

**Published:** 2021-05-13

**Authors:** Caitlin A. O’Connell, Andrea L. DiGiorgio, Alexa D. Ugarte, Rebecca S. A. Brittain, Daniel J. Naumenko, Sri Suci Utami Atmoko, Erin R. Vogel

**Affiliations:** 1 Department of Anthropology, Rutgers University, New Brunswick, USA; 2 The Writing Program, Princeton University, Princeton, USA; 3 Department of Anthropology, New York University, New York, USA; 4 Department of Anthropology, University of Colorado Boulder, Boulder, USA; 5 Institute of Behavioral Science, University of Colorado Boulder, Boulder, USA; 6 Faculty of Biology, Universitas Nasional, Jl.Sawo Manila, Jakarta, Indonesia; 7 Primate Research Center, Universitas Nasional, Jl. Sawo Manila, Jakarta, Indonesia

**Keywords:** Ecology, Evolution, Zoology, Ecology, Biomarkers

## Abstract

Pronounced temporal and spatial variation in the availability of food resources can produce energetic deficits in organisms. Fruit-dependent Bornean orangutans face extreme variation in fruit availability and experience negative energy and protein balance during episodes of fruit scarcity. We evaluate the possibility that orangutans of different sexes and ages catabolize muscle tissue when the availability of fruit is low. We assess variation in muscle mass by examining the relationship between urinary creatinine and specific gravity and use the residuals as a non-invasive measure of estimated lean body mass (ELBM). Despite orangutans having a suite of adaptations to buffer them from fruit scarcity and associated caloric deficits, ELBM was lower during low fruit periods in all age-sex classes. As predicted, adult male orangutans had higher ELBM than adult females and immatures. Contrary to expectation, flanged and unflanged males did not differ significantly in ELBM. These findings highlight the precarity of orangutan health in the face of rapid environmental change and add to a growing body of evidence that orangutans are characterized by unique metabolic traits shaped by their unpredictable forest environment.

## Introduction

The rainforests of Southeast Asia are characterized as challenging habitats for vertebrate frugivores^1^. In particular, these rainforests experience greater variation in annual fruit productivity than African and South American rainforests^2^. The island of Borneo is particularly limited, with lower overall fruit productivity compared to Sumatra^3,4^, and this low resource abundance has shaped the island’s fauna in various ways. For instance, Bornean mammal populations have smaller body sizes compared to their counterparts on Sumatra, Java, and the Malay/Thai Peninsula^5^. Larger than most other mammals on the island, orangutans cope with these conditions through unique behavioral, physiological, and morphological adaptations^6–8^. They experience extreme reductions in caloric intake when fruit is scarce, amounting to more than a 70% reduction in energy intake compared to high fruiting periods in some populations^7,9,10^. Behaviorally, orangutans are less gregarious than most haplorrhine primates, including all apes, and their semi-solitary nature has been attributed to their challenging foraging conditions^11^. During low fruit periods, orangutans spend less time traveling, have shorter active periods, and spend more time feeding^7^. Morphologically, Bornean orangutans have more robust mandibles for processing the tougher foods they consume compared to their Sumatran counterparts^8^ and other African apes^12^. Physiologically, orangutans have an extremely low basal metabolic rate and expend less energy than any other mammal that has been measured, except sloths^13^ and pandas^14^. In captivity, orangutans display a notable tendency to become obese^15–17^, and it has been argued that a propensity for storing fat during periods of fruit abundance which is then catabolized during lean periods sets orangutans apart from the other great apes^18^.

Despite this suite of adaptations to their challenging environment, low fruit periods are associated with declining ovarian hormone production, lower conception rates^18,19^, negative energy balance^20^, ketosis^9^, and negative protein balance^21,22^ in orangutans. Previous research has indicated that prolonged periods of low fruit availability on Borneo are associated with increased levels of δ^15^N in orangutan urine, indicating that somatic catabolism, or tissue wasting, was beginning to occur^22^. Thus, despite their enhanced tendency for fat storage^15–18,23^, orangutan fat reserves may not be sufficient and instead they may catabolize functional body tissues for energy. Here, we aim to evaluate this possibility by assessing whether variation in fruit availability predicts variation in estimated lean body mass among orangutans at the Tuanan Research Station.

Investigations of physiological variation via non-invasive means are integral to understanding the health, conservation status, and fitness consequences of behavioral and nutritional strategies in wild animals. While a wealth of research has evaluated the impact of ecological fluctuations on wild primate health through urinary and fecal indicators of energetic status, this research has been limited by an inability to assess body size and composition non-invasively^24^. Emery Thompson and colleagues^24^ proposed a solution to this challenge by measuring creatinine and specific gravity of urine collected from wild chimpanzees (*Pan troglodytes schweinfurthii*). Creatinine and specific gravity are both methods for assessing the water content of urine, but only creatinine is related to an individual’s muscle mass; individuals with greater muscle mass excrete more creatinine. Thus, the variation in creatinine that is unexplained by the variation in specific gravity represents an estimate of lean body mass^25,26^.

Following Emery Thompson et al.^24,25^, we examined creatinine and specific gravity of wild orangutan urine as a non-invasive estimate of lean body mass (ELBM). Due to significant reductions in caloric intake when fruit is scarce, we predicted that episodes of fruit scarcity would be associated with skeletal muscle wasting (e.g., lower ELBM). Based on body weight and size^27,28^, muscle mass is also expected to vary among the age-sex classes, with adult flanged male orangutans having the highest muscle mass, followed by adult unflanged males, then adult females, and then subadult individuals.

## Results

**Figure 1:**
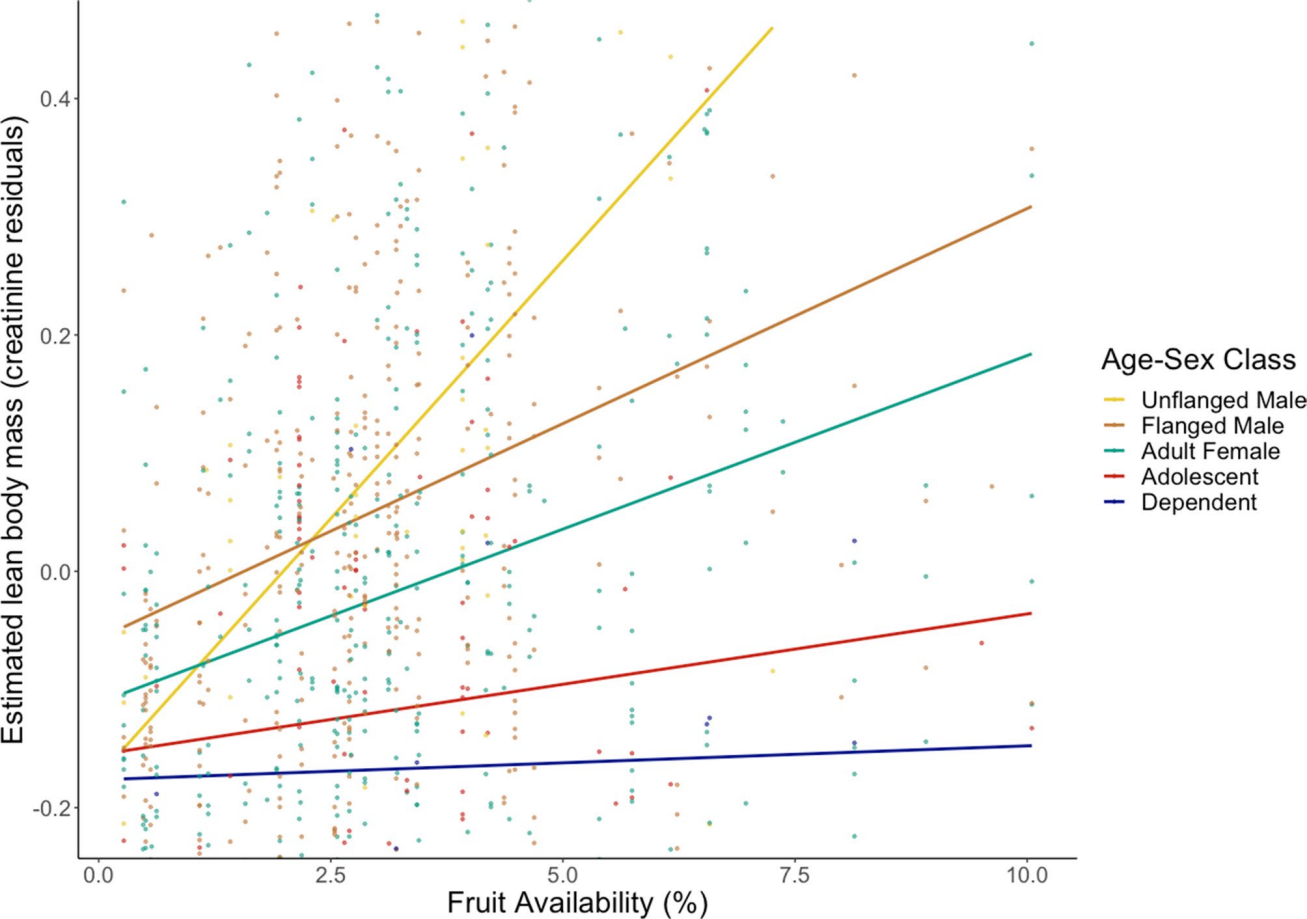
Linear regression lines describing the relationship between fruit availability and ELBM (residual creatinine) by age-sex class.

**Figure 2:**
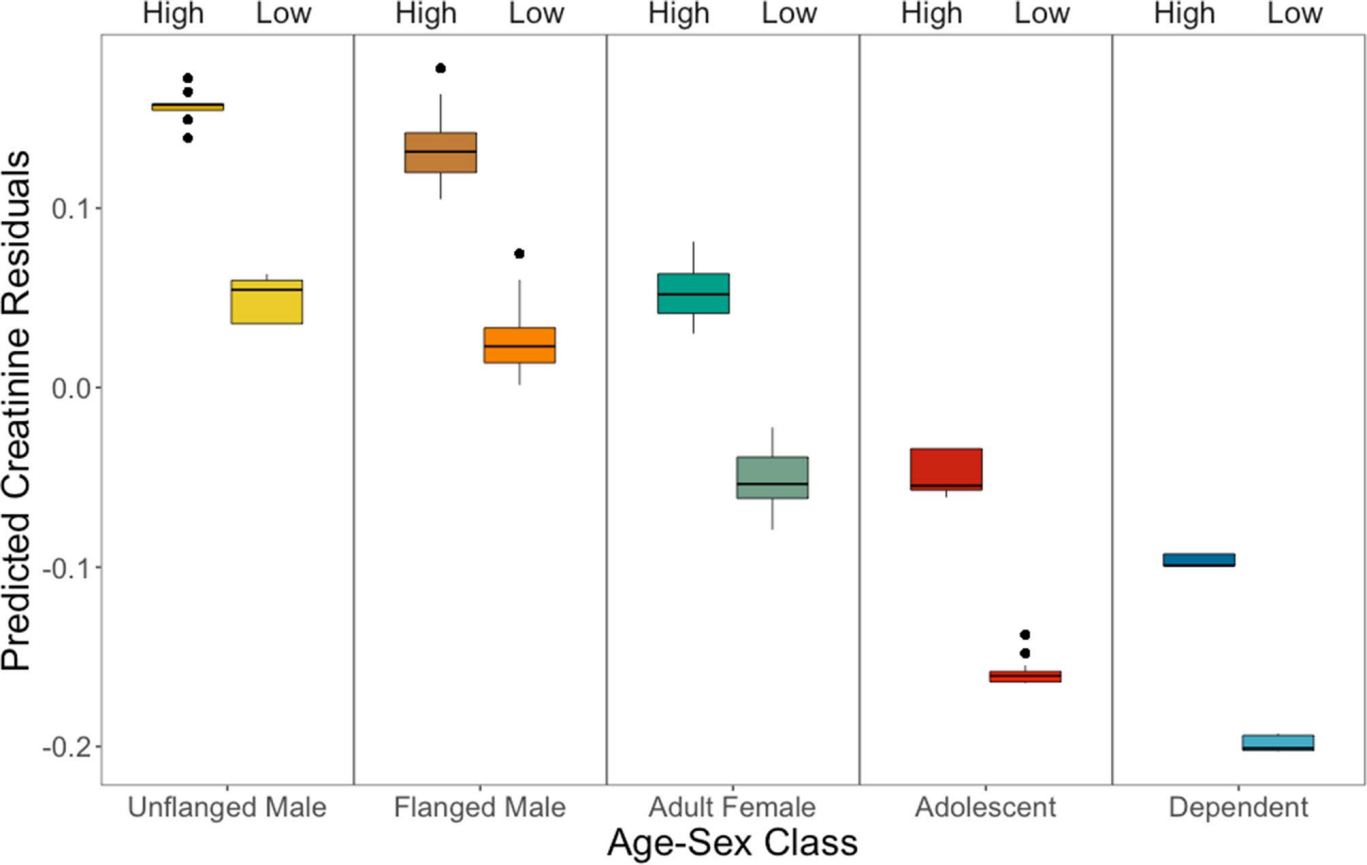
Predicted ELBM of each age-sex class using FAI as a binary predictor (high and low fruit, see S1 for GLMM results).

To detect changes in muscle mass, we measured the concentration of creatinine (range: 0.022–3.10 mg/ml) and the specific gravity of each urine sample (range: 1.003–1.055). ELBM was predicted by both age-sex class and FAI (Table 1). Episodes of fruit scarcity were associated with lower ELBMs (F_(5.094)_ = 13.6, *p* < 0.001); this lower estimated muscle mass measured during periods of fruit scarcity was consistent across all age-sex classes (Figs. 1, 2). This result held using FAI as a continuous variable or split into categorical high/low periods based on the 50% median (Fig. 2, see also S1).

**Table 1:** The relationship between ELBM, age-sex class, and FAI using GAMMs.

Baseline age-sex class	Model terms	β, (± SE)	t-stat, *p* value
Adult female	Intercept	− 0.02 (± 0.02)	t = − 0.90, *p* = 0.368
	Flanged male	0.07 (± 0.03)	t = 2.58**,** *p* = **0.01**
	Unflanged male	0.14 (± 0.05)	t = 2.61**,** *p* < **0.01**
	Adolescent	− 0.07 (± 0.04)	t = − 1.89, *p* = 0.059
	Dependent	− 0.16 (± 0.08)	t = − 1.48, *p* = 0.14
Flanged male	Intercept	0.05 (± 0.02)	t = 2.67, *p* < 0.01
	Unflanged male	0.07 (± 0.05)	t = 1.31, *p* = 0.191
	Adolescent	− 0.14 (± 0.04)	t = − 3.56**,** *p* < **0.001**
	Dependent	− 0.10 (± 0.08)	t = − 2.37**,** *p* = **0.018**
Unflanged male	Intercept	0.12 (± 0.05)	t = 2.44, *p* = 0.015
	Adolescent	− 0.21 (± 0.06)	t = − 3.48**,** *p* < **0.001**
	Dependent	− 0.25 (± 0.09)	t = − 2.81**,** *p* < **0.01**
Adolescent	Intercept	− 0.09 (± 0.03)	t = − 2.55, *p* = 0.011
	Dependent	− 0.05 (± 0.08)	t = − 0.55, *p* = 0.58

β coefficients presented are for the age-sex class in model term relative to the baseline age-sex class in the first column. Significant *p* values are highlighted in bold. Age-sex class (fixed effect), FAI (smoothed), and Individual ID (random effect) were included in each model (r^2^_adj_ = 0.09). FAI was a significant predictor in all models (F_(5.094)_ = 13.6, *p* < 0.0001).

ELBM varied among the age-sex classes in the general predicted pattern, with significantly greater ELBM for flanged males and adult unflanged males compared to all other age-sex classes (Table 1). ELBM for adult females, was not significantly different from adolescents and dependents although trended in the predicted direction (Fig. 1; Table 1).

## Discussion

Our results highlight that despite several well-documented adaptations to cope with unpredictable episodes of fruit scarcity in orangutans, lower ELBMs during low fruit periods suggest they still enter a critical state of muscle catabolism when fruit is scarce. Orangutans rely on less energy dense fallback foods when fruit availability is low^7^, and at several sites it has been demonstrated that they catabolize fat stores for energy during these periods^9,10,29^. Our findings extend previous evidence from another study site that orangutans enter into the early stages of a protein deficit after prolonged periods of fruit scarcity at Gunung Palung (West Kalimantan, Borneo). Gunung Palung is dominated by dipterocarp trees and, thus, experiences the masting phenomenon that is characteristic of these forest types^30,31^. Tuanan is a non-masting forest, with lower but more consistent fruit availability patterns than Gunung Palung^32^. Future research should compare patterns of muscle loss between these study sites to better understand variation in orangutan physiology in relationship to local ecologies.

The consistency of the suggested pattern of muscle wasting during low fruit periods across all age-sex classes raises questions about the impacts of muscle catabolism. Nutritional and energetic constraints have clearly shaped orangutan life history; they have the longest developmental period among apes^33^ and the slowest pace of reproduction among mammals^34^. This life history strategy may help buffer individuals against energy deficits by reducing the energetic requirements of growth or reproduction at a given time, which may reduce the need for muscle catabolism for energy. Future research should examine the long-term health and fitness impacts of more severe or frequent muscle wasting on individuals, particularly during sensitive windows such as juvenility or lactation.

We also predicted that there would be general differences in muscle mass among the age-sex classes. In chimpanzees, ELBM is highest in adult males, followed by sub-adult males, then adult females and sub-adult females^24^. Thus, we predicted a similar directional difference in ELBM for orangutans. In our study, ELBM differed among the age-sex classes in the expected pattern with some exceptions. Both adult male classes had higher ELBM than adult females and immature classes, but adult females did not differ from immature orangutans. There was no significant difference between independent immatures and dependent immatures in our sample. Figures 1 and 2 suggest that adult females trend towards greater ELBM than both classes of immatures, and we cannot rule out that the smaller sample size of the two immature groups (22 samples for dependents and 113 samples for adolescents, compared to 511 for adult females) obscured the differences. While it is less surprising that adult females and adolescent females did not show significant differences in ELBM, it is reasonable to expect a profound difference between adults and dependent individuals. Newborn human babies have high creatinine levels^35^, as do chimpanzees under the age of 3^24^. While no individuals in our sample were under the age of 3.5, it is possible that immature kidney functioning leading to reduced glomerular filtration^36^ caused higher creatinine levels in these young orangutans.

We also did not find a difference in ELBM between flanged and unflanged males. Flanged and unflanged male orangutans, while both fully sexually mature, have different appearances and body masses, with adult flanged males ranging from 74 to 90 kg^27,28^ and unflanged males 40.5 kg on average at one Bornean site (this average for unflanged males may be low as it comes from a disturbed forest site where orangutans tend to be thinner)^28^. These two male morphs differ in androgen levels^37–39^, with flanged males having higher levels of testosterone and DHT, which should promote muscle growth^40^. Thus, we expected substantial differences in ELBM between these two classes. It has been shown that male chimpanzees lose muscle mass as part of the ageing process. While it is possible that at least some of the flanged males in our sample were of advanced age, it is exceedingly difficult to track adult male orangutans over the long time periods to obtain an accurate estimate of age^41^, so we were unable to account for individual age in our study. Additionally, our sample size for unflanged males (53 samples among 15 individuals) was small compared to that of flanged males (431 samples among 31 individuals). Tuanan is characterized by a greater ratio of flanged relative to unflanged males, which is also true at nearby Sabangau^39^, but differs from a similar habitat site in Sumatra^42^. It is possible that with a more robust sampling of unflanged males this result could change, however obtaining a more robust sample at Tuanan would be challenging given the infrequent encounter rate for unflanged males. However, if indeed these two morphs do not differ in lean muscle mass, the difference in overall body mass between them would be the result, perhaps, of profoundly higher fat deposition in flanged males and/or a lower basal metabolic rate in the larger flanged males^43^. Male bimaturism in orangutans remains poorly understood, and these findings raise new questions about the eco-physiological differences between them.

These findings emphasize the ecological challenges that the critically endangered Bornean orangutan faces. Borneo is heavily impacted by El Niño events, and local climate shifts due to land conversion exacerbate the susceptibility of forests to fires during these periods^44^. As global climate change threatens to amplify El Niño events^45^, droughts and wildfires may worsen, having detrimental impacts on orangutan habitat and their food supply. Simple methods like the one used here will allow for better tracking of the health of Bornean orangutan populations. Long-term non-invasive monitoring of orangutan health status is critical, as it will provide insight into the effectiveness of current conservation practices and the types of new protections that must be implemented to protect vulnerable individuals and populations. These findings suggest that access to fruit is critical for orangutans, and emphasize the need for careful conservation planning, including thorough plant species surveys in forests being considered for the release of rehabilitants or translocated orangutans. The establishment and maintenance of forest corridors that connect fruit-poor fragments with more productive forest areas is imperative for orangutan survival. As demonstrated by variation among the age-sex classes in the relationship between muscle wasting and FAI, individual variation in vulnerability to different ecological challenges must be considered as part of effective conservation plans.

## Methods

Data were collected at Tuanan Research Station in Central Kalimantan, Indonesia from 2009 to 2017. The study site contains only wild orangutans, with no ex-captives or translocated individuals released at the study site. A fruit abundance index (FAI) was determined each month as the percent of stems with a diameter at breast height (dbh) greater than 10 cm that were fruiting (n = 2400 stems) within phenology plots^6^. The phenology plots cover 2.3 ha within the 900 ha research site and are spread across the home ranges of the most heavily sampled orangutans in the dataset. Age-sex classes were determined by developmental status and sex^7^, and included adult females N = 32; adult flanged males N = 31; adult unflanged males N = 15; adolescent males and females, which includes both independent and semi-independent immatures N = 11 (weaned and/or older sibling animals of smaller body size than adults—independents rarely travel with mother, semi-independent travel with mother most of the time); and dependent male and female offspring N = 11 (clinging or non-clinging unweaned animals sleeping in mother’s nest and in daily association with mother).

Urine was collected from first-morning voids during nest-nest focal follows and kept on ice in a thermos. Upon return to the research camp, SG was measured using a digital refractometer (Atago PAL-10S), and samples were then frozen at − 20 °C. Samples were shipped to Rutgers University on dry ice and stored at − 80 °C until analyzed for creatinine following Emery Thompson et al.^24,25^. We followed previously validated methods to estimate lean body mass from creatinine adjusted by SG in spot urine samples^24,25^. We first removed all overly dilute samples with SG less than 1.003 (n = 14), resulting in 1,130 samples from 70 individuals that were used in our analyses (Table 2). Water has a SG of 1.0 and a creatinine level of 0.0 mg/ml, so we subtracted 1 from each SG reading and used the resulting values to calculate a global fit for creatinine levels against [(SG − 1) + (SG − 1)^2^] via linear regression forced through the origin (r^2^_adj_ = 0.812, *p* < 0.0001). We used both the linear and quadratic terms for specific gravity to account for the curvilinear relationship between creatinine and SG. The residuals from this regression represented the variation in creatinine unexplained by SG, indicating an estimate of lean body mass^25^. The residuals were used as the target in a generalized additive mixed model (GAMM) with FAI and age-sex class as fixed effects and individual ID as a random factor. Pairwise comparisons were achieved via the *relevel* function for age-sex class in the GAMM. A generalized linear mixed model (GLMM) with FAI (binned as High and Low) and age-sex class was used for visualization (Fig. 2, see S1 for results). All statistical analyses were run in R version 3.6.1^46^. R code is available at https://github.com/adigiorgio17/sg-cre-ou.

**Table 2:** Number of samples and summary statistics for each age-sex class.

Age-sex class	# Samples	Mean creatinine residual	SD	Median	IQR
Adult female	511	− 0.0167	0.385	− 0.0817	0.364
Flanged male	431	0.0508	0.314	0.00578	0.349
Unflanged male	53	0.107	0.297	0.0462	0.382
Adolescent	113	− 0.119	0.264	− 0.107	0.33
Dependent	22	− 0.166	0.296	− 0.241	0.174

### Ethical approval

This research followed all international, national, and institutional guidelines for the care and use of animals. This protocol was approved by the Institutional Animal Care and Use Committee of Rutgers, the State University of New Jersey, PROTO999900338. A CITES export permit was obtained to transport urine samples (permit #s:15497/IV/SATS-LN/2019, 22655/IV/SATS-LN/2017, 17/BKSDA.KALTENG-1/2015) from Indonesia.

## Supplementary Information

Supplementary Information

## Data Availability

The dataset analyzed for the current study are available from the authors upon reasonable request.
