# Supplementary material for: Wild Bornean orangutans experience muscle catabolism during episodes of fruit scarcity
Source: Sci Rep. 2021 May 13;11:10185. doi: 10.1038/s41598-021-89186-4 (PMC8119411; doi:10.1038/s41598-021-89186-4)
Supplement: Supplementary file 1 — Supplementary Information [file 41598_2021_89186_MOESM1_ESM.docx]

**Wild Bornean orangutans experience muscle catabolism during episodes of fruit scarcity**

Caitlin A. O’Connell, Andrea L. DiGiorgio, Alexa D. Ugarte, Rebecca S.A. Brittain, Daniel J. Naumenko, Sri Suci Utami Atmoko, Erin R. Vogel

**Supplement 1.**

Fixed coefficients of the GLMM predicting ELBM using FAI as a binary predictor (Figure 2), High Fruit Availability as comparative condition. In this model, Age-Sex class was a significant predictor (F_5, 56.93_ = 8.712, p = 3.478e-06) as was Fruit Availability (binary, F_1, 1104.89_ = 22.500, p = 2.376e-06).

| **Fixed Effects** | **Estimate** | **SE** | **df** | **t-value** | **P-value** |
| --- | --- | --- | --- | --- | --- |
| Adult Female | 0.0498 | 0.0245 | 27.663 | 2.030 | 0.0520 |
| Flanged Male | 0.1398 | 0.0256 | 64.942 | 5.454 | 8.23e-07 |
| Unflanged Male | 0.1368 | 0.0478 | 315.431 | 2.859 | 0.00453 |
| Dependent | -0.0782 | 0.0726 | 667.962 | -1.077 | 0.2821 |
| Adolescent | -0.0446 | 0.0376 | 131.708 | -1.184 | 0.2386 |
| Low Fruit Avail. | -0.1064 | 0.0224 | 1104.886 | -4.743 | 2.38e-06 |
